# Relationship between type 1 diabetes and autoimmune diseases in european populations: A two-sample Mendelian randomization study

**DOI:** 10.3389/fgene.2024.1335839

**Published:** 2024-09-16

**Authors:** Weidong Xie, Haojie Jiang, Yao Chen, Zhaojie Yu, Yaoyu Song, Huanhao Zhang, Sen Li, Shaoliang Han, Naxin Liu

**Affiliations:** ^1^ Department of Gastrointestinal Surgery, The First Affiliated Hospital of Wenzhou Medical University, Wenzhou, China; ^2^ Department of Medical Oncology, Sir Run Run Shaw Hospital, School of Medicine, Graduate School, Zhejiang University, Hangzhou, China; ^3^ The First School of Medicine, School of Information and Engineering, Wenzhou Medical University, Wenzhou, China; ^4^ School of Public Health and Management, Wenzhou Medical University, Wenzhou, China; ^5^ School of Basic Medicine, Wenzhou Medical University, Wenzhou, China

**Keywords:** systemic lupus erythematosus (SLE), rheumatoid arthritis (RA), inflammatory bowel disease (IBD), type 1 diabetes(T1D), Mendelian randomization(MR)

## Abstract

**Background:**

Previous studies have suggested an association between Type 1 diabetes (T1D) and autoimmune diseases (AIDs), but the causal relationship remains unclear. Therefore, this study utilizes publicly available Genome-Wide Association Studies (GWAS) databases and employs a two-sample Mendelian Randomization (MR) approach to explore the causal relationships between T1D and systemic lupus erythematosus (SLE), rheumatoid arthritis (RA), and inflammatory bowel disease (IBD).

**Methods:**

Summary GWAS data for T1D, SLE, RA, and IBD were downloaded from open GWAS databases and the International Inflammatory Bowel Disease Genetics Consortium (IIBDGC). We employed a series of methods to select instrumental variables closely related to T1D. To enhance the reliability of our conclusions, we applied multiple robust analytical methods, with the inverse variance weighted (IVW) method as the primary approach. Validation and meta-analysis were conducted using the FinnGen consortium. Additionally, we assessed heterogeneity, pleiotropy, and sensitivity to ensure the robustness of our conclusions.

**Results:**

A potential causal association was found between T1D and SLE (OR = 1.37, 95% CI = 1.26 – 1.49, P < 0.001), which was further confirmed by meta-analysis. Similarly, a potential causal association was found between T1D and RA (OR = 1.32, 95% CI = 1.17 – 1.50, P < 0.001), and this was also confirmed by meta-analysis. Although the association between T1D and IBD showed P < 0.05, the leave-one-out test did not pass, and further meta-analysis indicated no significant statistical association between them.

**Conclusion:**

Our study reveals the relationships between T1D and three clinically common autoimmune diseases (SLE, RA, and IBD). This research supplements previous studies and provides a reference for future clinical work.

## Introduction

Autoimmune diseases (AIDs) are a group of complex chronic diseases of unknown etiology characterized by defects in immune tolerance. Common autoimmune diseases include systemic lupus erythematosus (SLE), rheumatoid arthritis (RA), and inflammatory bowel disease (IBD) (Gao et al., 2021). In the United States, autoimmune diseases are one of the leading causes of death among young and middle-aged women (Cooper and Stroehla, 2003). Additionally, because these conditions are often lifelong, they impose a significant burden on both society and individuals (Roberts and Erdei, 2020; Rose, 2016).

Type 1 diabetes (T1D) is an autoimmune disease characterized by insulin deficiency and resultant hyperglycemia (DiMeglio et al., 2018). It commonly occurs in individuals aged 10–14 years (DiMeglio et al., 2018; Maahs et al., 2010). The current understanding is that its pathogenesis may be related to a T-cell-mediated autoimmune process targeting pancreatic β-cells, with its incidence increasing globally (Vehik and Dabelea, 2011). The relationship between T1D and autoimmune diseases has long been both intriguing and perplexing. Clinically, it has been observed that patients with T1D often have other autoimmune diseases, such as dermatological and rheumatic conditions (Popoviciu et al., 2023). Research indicates that T1D and other autoimmune diseases may share certain pathways or genes (Szymczak et al., 2021). However, the causal relationship between T1D and other autoimmune diseases remains unclear.

Observational studies may struggle to correctly determine causality or may produce spurious associations due to the presence of some unavoidable biases (Boyko, 2013). Therefore, in this study, we use Mendelian Randomization (MR) to further investigate the causal relationship between T1D and three clinically common autoimmune diseases (SLE RA and IBD). Mendelian Randomization uses genetic variation as an instrumental variable for the exposure, thereby determining the causal relationship between the exposure and the outcome (Davey Smith and Hemani, 2014; Yarmolinsky et al., 2018). This method can avoid reverse causation and potential confounding biases, making the results more convincing (Zoccali et al., 2006).

## Materials and method

### Study Design

**FIGURE 1:**
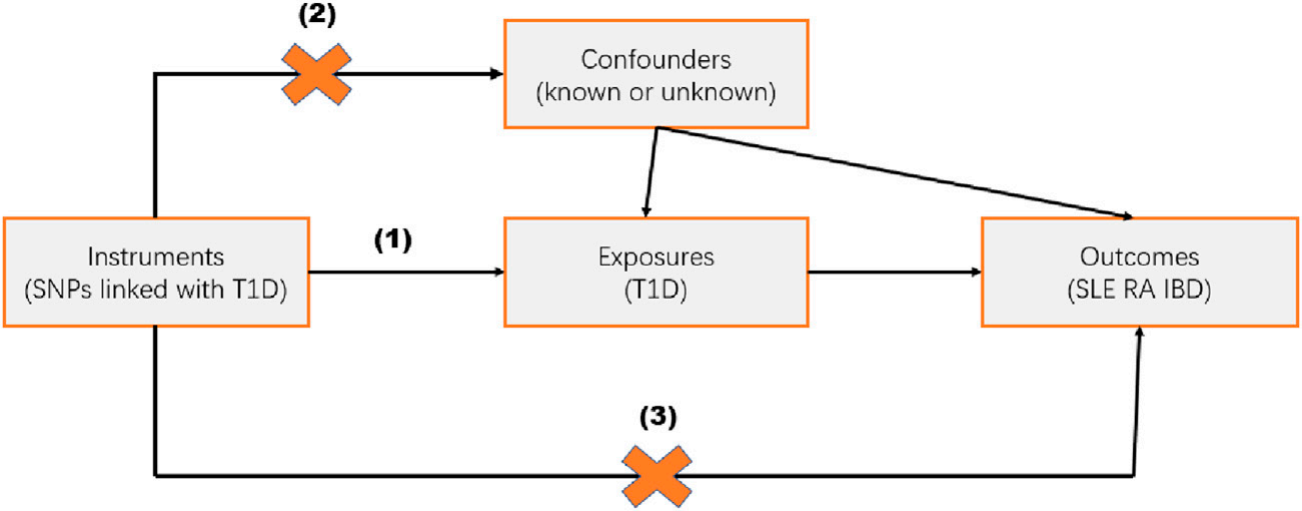
Study Design. Mendelian randomization studies are based on three assumptions: (1) the instrumental variable (IV) is strongly associated with the exposure; (2) the IV is independent of known or unknown confounders; (3) the IV influences the outcome only through the exposure.

Mendelian Randomization (MR) studies typically use single nucleotide polymorphisms (SNPs) as instrumental variables (IVs). Conducting an MR analysis requires meeting the following three assumptions (Figure 1): (1) the IVs are strongly associated with the exposure; (2) the IVs are not associated with potential confounders; (3) the IVs influence the outcome only through the exposure. The data used in this study are publicly available and free, thus no further ethical review or patient consent is required.

### Data sources

To ensure the robustness of the results and the generalizability of the conclusions, we selected databases from two different sources for each outcome. Details of the data are shown in Table 1.

**TABLE 1:** Data sources.

Phenotype	Data source	Sample size (cases/controls)
Exposure		
T1D	Vincenzo Forgetta et al	9266/15574
outcome		
SLE	James Bentham et al	5201/9066
SLE	FinnGen	538/213145
RA	Yukinori Okada et al	14361/43923
RA	FinnGen	6236/147221
IBD	IIBDGC	31665/33977
IBD	FinnGen	5673/213119

SNPs related to T1D were obtained from a large Genome-Wide Association Studies (GWAS) study, which included 9,266 cases and 15,574 controls (Forgetta et al., 2020).

SNPs related to SLE were obtained from a large GWAS study that included 5,201 cases and 9,066 controls (Bentham et al., 2015). Moreover, SLE data from the Finnish database (FinnGen) included 538 cases and 213,145 controls.

SNPs related to RA were obtained from a large GWAS study that included 14,361 cases and 43,923 controls (Okada et al., 2014). Moreover, RA data from the Finnish database (FinnGen) included 6,236 cases and 147,221 controls.

SNPs related to IBD were obtained from a study by the International Inflammatory Bowel Disease Genetics Consortium (IIBDGC), which is the largest genetic database for IBD globally. This study included 31,665 cases and 33,977 controls after quality control (QC) (Liu et al., 2015). In addition, IBD data from the Finnish database (FinnGen) included 5,673 cases and 213,119 controls.

When multiple GWAS databases were available, we prioritized those with larger sample sizes, more SNPs, and greater citation frequency by researchers.

### Meta-analysis

To validate the robustness of the results, we further verified the outcomes within the FinnGen consortium. Subsequently, we conducted a meta-analysis to further ascertain the relationship between T1D and the different autoimmune diseases. In the meta-analysis, a random effects model was used if heterogeneity (*p* < 0.05) was present; if no heterogeneity was detected (*p* > 0.05), a fixed effects model was employed.

### Selection of genetic instruments

To ensure adherence to the assumptions of Mendelian Randomization, we selected instrumental variables based on the following criteria (Gagliano Taliun and Evans, 2021): we used a threshold of *p* < 5 × 10^ -8 as the primary filter to ensure that the SNPs were strongly associated with the characteristics of T1D. Moreover, we excluded SNPs in linkage disequilibrium (LD) (R^ 2 < 0.001, clumped at 10,000 kb). We also calculated the F-statistic to test for bias due to weak instruments, using the formula: F = β ^2/se^ 2 (Wang et al., 2022; Zhao et al., 2023; Li and Martin, 2002). An F-statistic greater than 10 was required to minimize bias from weak instruments (Burgess et al., 2011).

### Statistical analysis

In this study, MR analysis was conducted using the TwoSampleMR package (version 0.5.6) and R software (version 4.2.1) (Yavorska and Burgess, 2017). Meta-analysis was performed using Review Manager (version 5.4). The primary analysis method was the Inverse Variance Weighted (IVW) approach, which combines the Wald ratio estimates of each SNP to produce a pooled estimate (Pierce and Burgess, 2013). Supplementary analyses included: (1). Weighted Median (Bowden et al., 2016). This method can provide consistent estimates of causal effects even if up to 50% of the instruments are invalid; (2). MR Egger (Bowden et al., 2015). This method offers consistent estimates of pleiotropy even if all instruments are invalid; 3. MR-PRESSO (Verbanck et al., 2018). This method identifies outliers with horizontal pleiotropy and is most effective when less than 50% of the instruments exhibit horizontal pleiotropy. Cochran’s Q test was used to detect heterogeneity (Greco et al., 2015). The intercept test from MR Egger regression was employed to evaluate horizontal pleiotropy (Bowden et al., 2015).

## Results

### Selection of instrumental variables

We selected IVs based on the criteria outlined above. Ultimately, we identified 44 SNPs to be used as IVs for T1D. Moreover, all F-statistics were greater than 10, indicating the absence of weak instrument bias (Supplementary Table S1).

### Relationship between T1D and SLE

**FIGURE 2:**
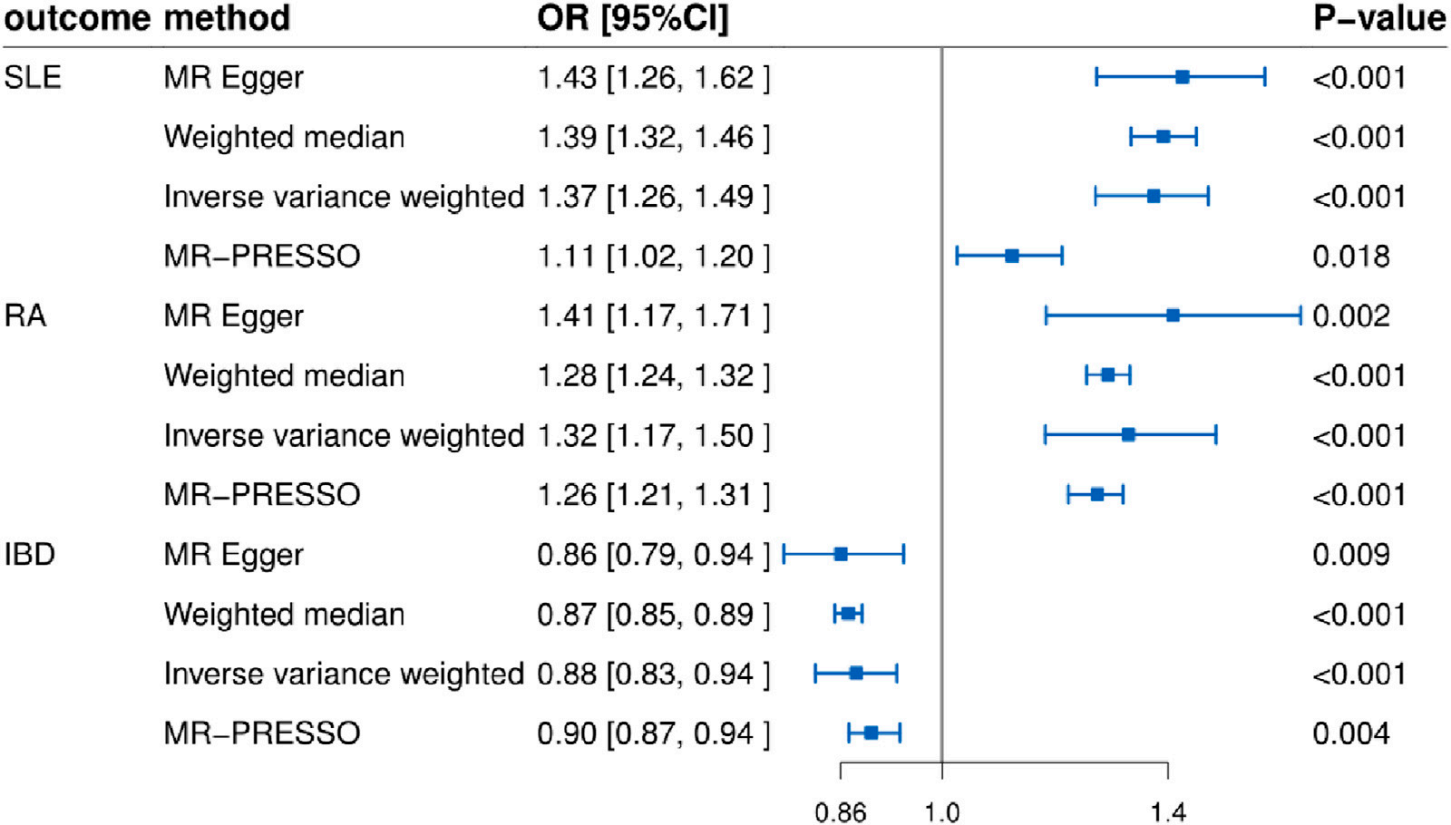
Forest plots showing the relationship between T1D and SLE, RA, and IBD (non-FinnGen databases).

**FIGURE 3:**
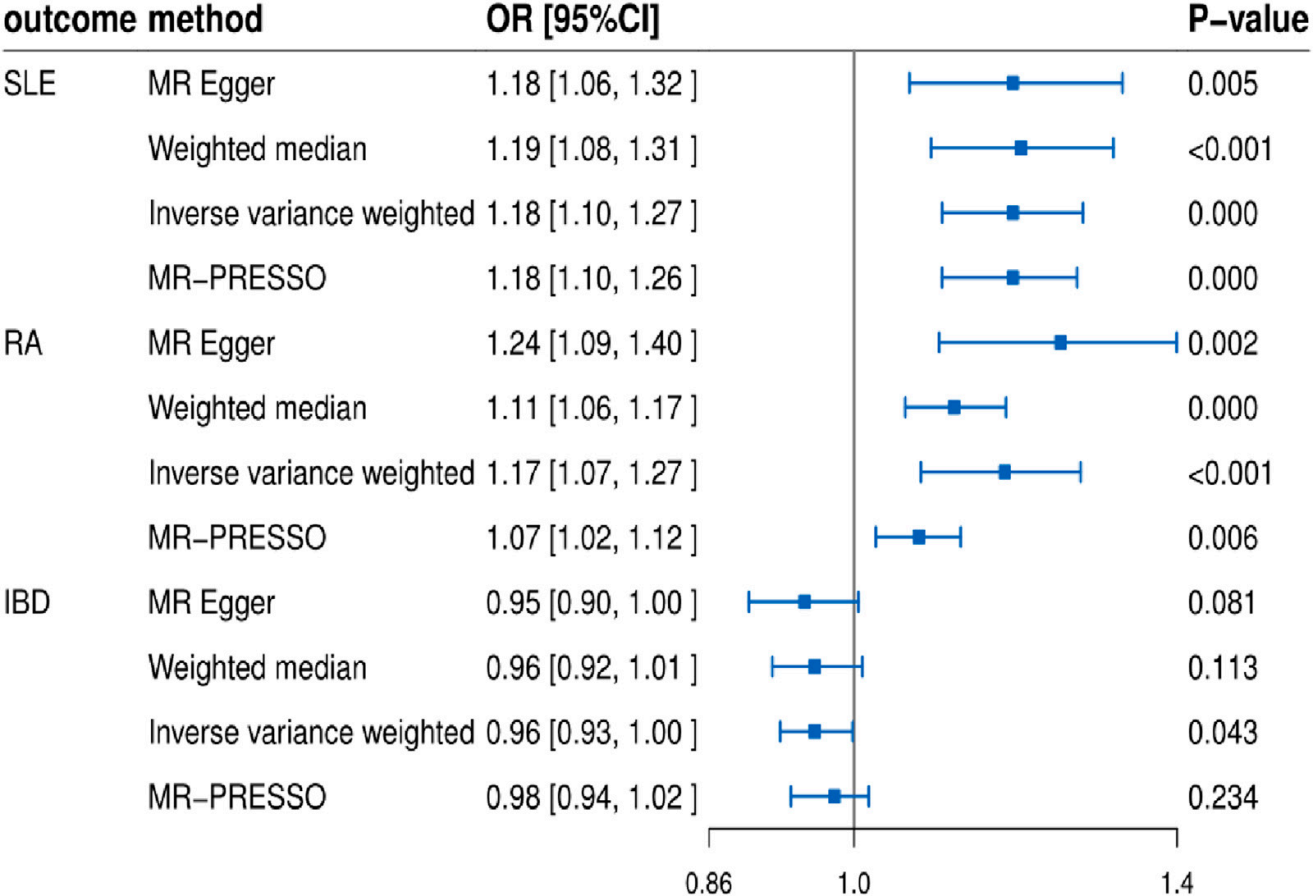
Forest plots showing the relationship between T1D and SLE, RA, IBD (FinnGen database).

**FIGURE 4:**
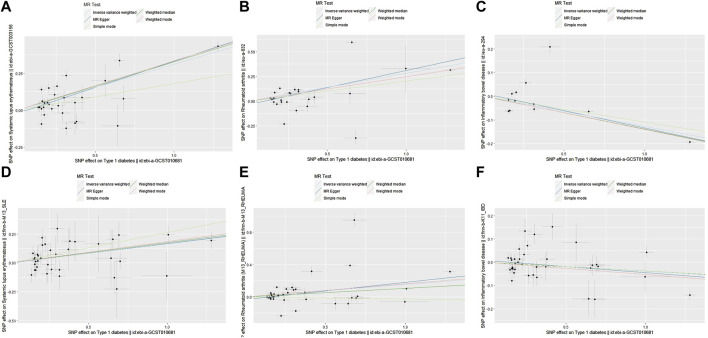
Scatter plots showing the relationship between T1D and SLE, RA, IBD. **(A)** T1D and SLE (non-FinnGen databases); **(B)** T1D and RA (non-FinnGen databases) **(C)** T1D and IBD (non-FinnGen databases); **(D)** T1D and SLE (FinnGen database) **(E)** T1D and RA (FinnGen database); **(F)** T1D and IBD (FinnGen database).

**FIGURE 5:**
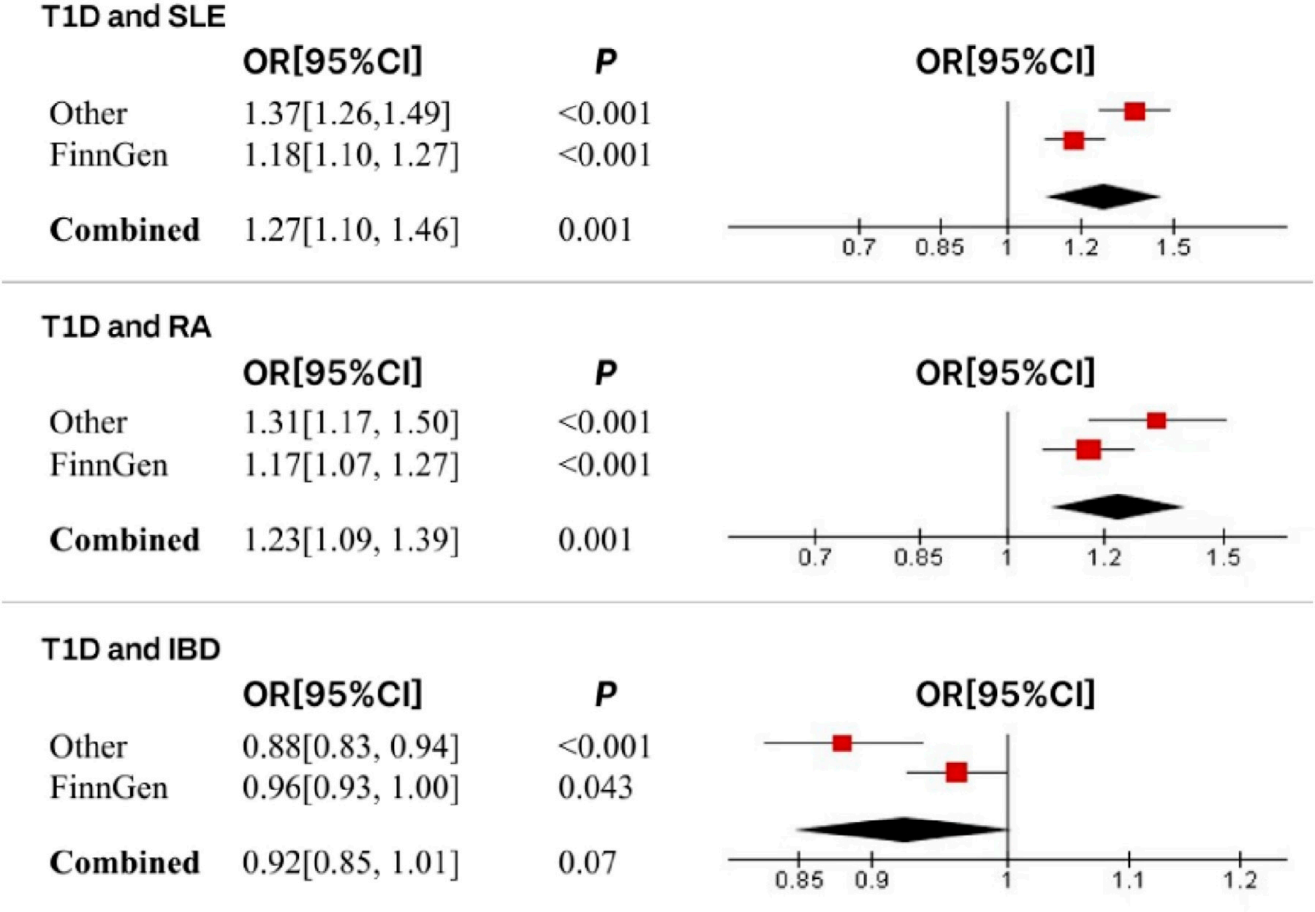
Meta-analysis results and forest plots for the relationship between T1D and SLE, RA, and IBD.

In this study, we found that T1D exhibited a positive association with SLE (OR = 1.37, 95% CI = 1.26–1.49, *p* < 0.001). This result remained robust even after removing outliers using the MR-PRESSO method (OR = 1.11, 95% CI = 1.02–1.20, *p* = 0.018) (Figures 2, 4; Table 2). Within the FinnGen consortium, T1D continued to show a positive association with SLE (OR = 1.18, 95% CI = 1.10–1.27, *p* < 0.001) (Figures 3, 4; Table 2). Meta-analysis further confirmed the relationship between the two (OR = 1.27, 95% CI = 1.10–1.46, *p* = 0.001) (Figure 5).

**TABLE 2:** MR analysis results for T1D with SLE, RA, and IBD.

Outcome	Data source	Methods	OR	95%CI	P-Value
SLE	James Bentham et al	MR-Egger	1.43	1.26–1.62	P<0.001
		Weighted median	1.39	1.32–1.46	P<0.001
		IVW	1.37	1.26–1.49	P<0.001
		MR-PRESSO	1.11	1.02–1.20	P = 0.018
SLE	FinnGen	MR-Egger	1.18	1.06–1.32	P = 0.005
		Weighted median	1.19	1.08–1.31	P<0.001
		IVW	1.18	1.10–1.27	P<0.001
		MR-PRESSO	1.18	1.10–1.26	P<0.001
RA	Yukinori Okada et al	MR-Egger	1.41	1.17–1.71	P = 0.002
		Weighted median	1.28	1.24–1.32	P<0.001
		IVW	1.32	1.17–1.50	P<0.001
		MR-PRESSO	1.26	1.21–1.31	P<0.001
RA	FinnGen	MR-Egger	1.24	1.09–1.40	P = 0.002
		Weighted median	1.11	1.06–1.17	P<0.001
		IVW	1.17	1.07–1.27	P<0.001
		MR-PRESSO	1.07	1.02–1.12	P = 0.006
IBD	IIBDGC	MR-Egger	0.86	0.79–0.94	P = 0.009
		Weighted median	0.87	0.85–0.89	P<0.001
		IVW	0.88	0.83–0.94	P<0.001
		MR-PRESSO	0.90	0.87–0.94	P = 0.004
IBD	FinnGen	MR-Egger	0.95	0.90–1.00	P = 0.081
		Weighted median	0.96	0.92–1.01	P = 0.113
		IVW	0.96	0.93–1.00	P = 0.043
		MR-PRESSO	0.98	0.94–1.02	P = 0.234

### Relationship between T1D and RA

In our study, we found a positive association between T1D and RA (OR = 1.32, 95% CI = 1.17–1.50, *p* < 0.001). This positive association persisted even after removing outliers using the MR-PRESSO method (OR = 1.26, 95% CI = 1.21–1.31, *p* < 0.001) (Figures 2, 4; Table 2). This conclusion was also validated using data from the FinnGen consortium (OR = 1.17, 95% CI = 1.07–1.27, *p* < 0.001) (Figures 3, 4; Table 2). Meta-analysis further confirmed the relationship between T1D and RA (OR = 1.23, 95% CI = 1.09–1.39, *p* = 0.001) (Figure 5).

### Relationship between T1D and IBD

In this study, our analysis indicated a negative association between T1D and IBD (OR = 0.88, 95% CI = 0.83–0.94, *p* < 0.001) (Figures 2, 4; Table 2). However, it is noteworthy that the leave-one-out analysis suggested that this relationship might be disproportionately influenced by a single SNP, indicating potential bias in the results (Supplementary Figure S1). In the FinnGen consortium, a similar trend was observed between T1D and IBD (OR = 0.96, 95% CI = 0.93–1.00, *p* = 0.043) (Figures 3, 4; Table 2), but the leave-one-out analysis still indicated potential bias (Supplementary Figure S1). Meta-analysis showed no significant statistical difference (OR = 0.92, 95% CI = 0.85–1.01, *p* = 0.07) (Figure 5).

### MR sensitivity analysis results

The MR Egger regression intercept indicated limited evidence of horizontal pleiotropy (Table 3). For T1D and SLE, as well as T1D and RA, the leave-one-out analysis demonstrated that the causal associations were not unduly influenced by any single SNP. However, the leave-one-out analysis suggested that the causal association between T1D and IBD might be disproportionately affected by a single SNP. Heterogeneity tests for each group are presented in Table 3. The forest plots and volcano plots provide a more visual representation of the heterogeneity (Supplementary Figures S2, S3).

**Table 3:** Heterogeneity and Horizontal Pleiotropy Test Results

Outcome	Pleiotropy		Heterogeneity		Data source
	Intercept	P	Q	P	
SLE	−0.022	P = 0.37	139	P < 0.001	James Bentham et al
SLE	−0.002	P = 0.94	47	P = 0.08	FinnGen
RA	−0.032	P = 0.39	720	P < 0.001	Yukinori Okada et al
RA	−0.033	P = 0.21	543	P < 0.001	FinnGen
IBD	0.012	P = 0.58	161	P < 0.001	IIBDGC
IBD	0.007	P = 0.54	116	P < 0.001	FinnGen

## Discussion

In this study, we used MR to evaluate the causal relationships between T1D and several clinically common autoimmune diseases. Our research indicated that genetic susceptibility to T1D was associated with an increased risk of both SLE and RA, but not with IBD.

T1D is a complex chronic disease that is often found to co-occur with other autoimmune diseases in clinical settings (Zeglaoui et al., 2010; Çetın et al., 2013). A study from Sweden involving 3,093 participants demonstrated a significant association between T1D and RA (OR = 4.9, 95% CI = 1.8, 13.1), which is consistent with our findings (Liao et al., 2009). Although previous views suggested that T1D is not an independent risk factor for RA (Popoviciu et al., 2023), our analysis indicated a possible causal relationship, and Zhernakova et al. have also identified shared genetic risk loci between T1D and RA (Zhernakova et al., 2007). Similarly, a study based on the HealthFacts database showed that patients with T1D are more likely to develop SLE, another rheumatic disease (1325/158865) (Bao et al., 2019), compared to an incidence rate of approximately 23.2 per 100,000 in the general North American population (Popoviciu et al., 2023). Additionally, both RA and SLE are more commonly co-morbid in female T1D patients than in males (Bao et al., 2019; Bao et al., 2018). Therefore, clinicians should be vigilant in preventing rheumatic diseases in T1D patients, especially in females, to reduce potential risks and economic burdens on patients.

Although SLE and RA are distinct diseases, they both fall under the category of rheumatic diseases. Previous researches have shown that RA and SLE share familial aggregation (Cardenas-Roldan et al., 2013), genetic (Cui et al., 2013; Orozco et al., 2011; Marquez et al., 2017), molecular mechanisms (Higgs et al., 2011), and targeted therapies (Petitdemange et al., 2020), which might partially explain why both are associated with T1D. Studies have indicated that the interleukin two receptor subunit alpha (IL2RA) gene is closely related to the onset of T1D (Pahkuri et al., 2023), and IL2RA is also implicated in the pathogenesis of SLE and RA (Gorji et al., 2019; van Steenbergen et al., 2015). Our study also identified that mutations in the IL2RA gene (rs12722495) might contribute to the associations observed between these conditions.

The causal relationship between T1D and IBD has long been debated. A study from Denmark indicated a significant association between T1D and IBD (Halling et al., 2017). However, other studies have found no significant association between the two (Cohen et al., 2008; Lu et al., 2020), which aligns with our findings. Although our results confirm some previous clinical studies, several important points deserve attention: Firstly, T1D commonly occurs in individuals aged 10–14 years (DiMeglio et al., 2018; Maahs et al., 2010), whereas IBD tends to develop in young and middle-aged adults (He et al., 2022). This study targeted an adult population. For the pediatric population, a study involving 1,200 cases found an association between IBD and diabetes (Kappelman et al., 2011). Additionally, research from Austria and Germany observed a higher incidence of IBD in children with T1D compared to their age-matched peers (Jasser-Nitsche et al., 2021). Therefore, the relationship between T1D and early-onset IBD in children warrants further investigation. Secondly, although our study did not find a statistically significant relationship between T1D and IBD, the P-value was close to 0.05, suggesting a potential negative association trend. Previous studies have shown that protein tyrosine phosphatase non-receptor type 22 (PTPN22) plays an opposing role in Crohn’s disease compared to T1D (Barrett et al., 2008). Research indicates that PTPN22 knockdown activates inflammatory signaling pathways, leading to Crohn’s disease (Spalinger et al., 2013). Conversely, PTPN22 knockdown does not increase the risk of T1D and may even confer protective effects (Zheng and Kissler, 2013). Similarly, risk alleles for T1D, such as Interleukin 27 (IL-27), Interleukin 10 (IL-10), and interleukin-18 receptor 1 (IL18RA), have been found to prevent Crohn’s disease. Major histocompatibility complex (MHC) alleles strongly associated with T1D risk also appear to prevent both Crohn’s disease and ulcerative colitis (Wang et al., 2010). In contrast, PTPN22 is implicated in promoting the development of RA and SLE (Liao et al., 2009; Deng and Tsao, 2010). This intriguing phenomenon may relate to the “direction” of genetic variants: if a variant is associated with multiple autoimmune diseases but in opposite directions, it is more likely to be involved in pathways related to immune function, exhibiting contrasting characteristics (Wang et al., 2010).

Compared with traditional research methods, our study has several advantages. Firstly, we used Mendelian Randomization to evaluate the relationship between T1D and other autoimmune diseases. This method significantly reduces the impact of confounding bias and reverse causation. Secondly, our instrumental variables were derived from large-scale GWAS, providing reliable and robust SNP data, which helps avoid bias from weak instruments. Additionally, we conducted meta-analyses to further assess the reliability of our results.

However, our study has some limitations. Firstly, although we used various methods to analyze horizontal pleiotropy, we cannot entirely rule out the presence of potential horizontal pleiotropy. Secondly, Mendelian Randomization itself may face new issues, such as the “winner’s curse” (Yarmolinsky et al., 2018). Lastly, our study was limited by its focus on a specific ethnic group, so the conclusions might not be generalizable to other populations.

## Conclusion

In summary, our study further substantiated the causal relationships between T1D and both RA and SLE, while no association was found between T1D and IBD. These findings suggested that in managing patients with T1D, attention should be given to preventing RA and SLE to reduce potential complications and economic burdens for patients.

## Data Availability

The original contributions presented in the study are included in the article/Supplementary Material, further inquiries can be directed to the corresponding authors.
