# Supplementary material for: Relationship between type 1 diabetes and autoimmune diseases in european populations: A two-sample Mendelian randomization study
Source: Front Genet. 2024 Sep 16;15:1335839. doi: 10.3389/fgene.2024.1335839 (PMC11439667; doi:10.3389/fgene.2024.1335839)
Supplement: Supplementary file 2 [file Image1.pdf]

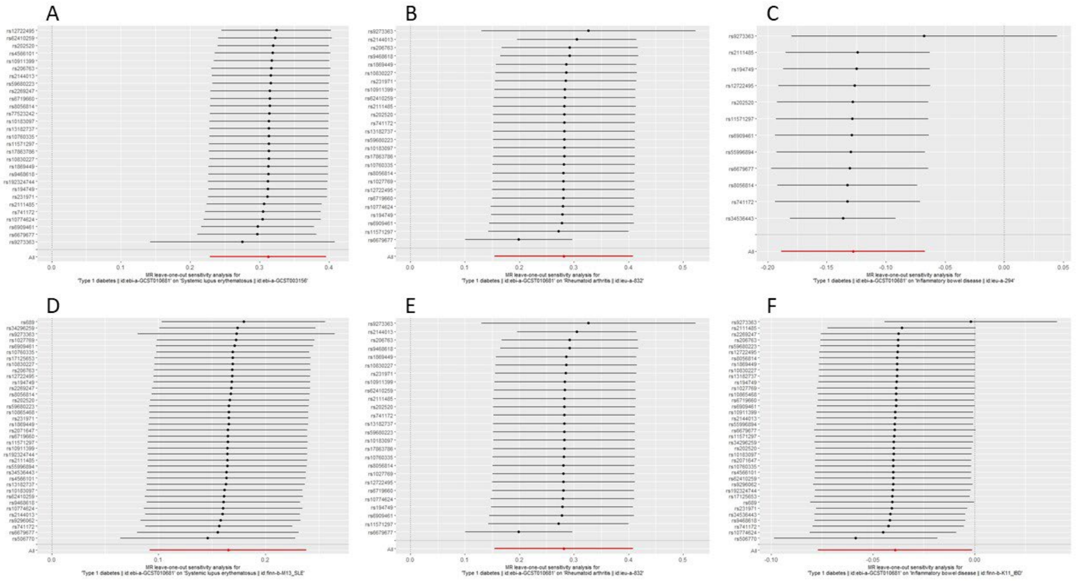

Supplementary Figure 1: Leave-one-out sensitivity test

A: T1D and SLE (non-FinnGen); B: T1D and RA (non-FinnGen);

C: T1D and IBD (non-FinnGen); D: T1D and SLE (FinnGen);

E: T1D and RA (FinnGen); F: T1D and IBD (FinnGen)

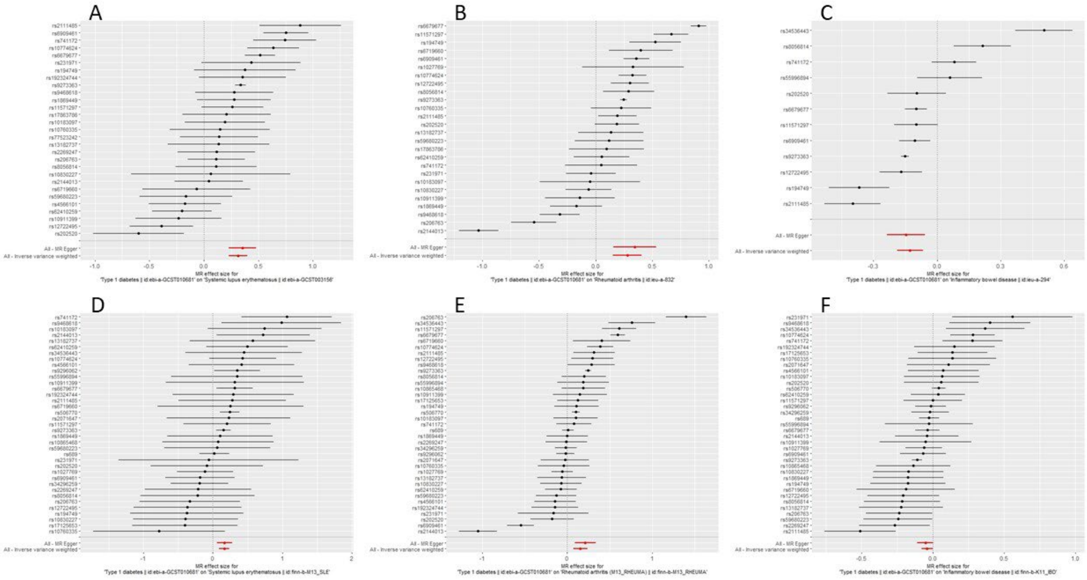

Supplementary Figure 2: Forest plot of the relationship between T1D and SLE, RA, IBD

A: T1D and SLE (non-FinnGen); B: T1D and RA (non-FinnGen);

C: T1D and IBD (non-FinnGen); D: T1D and SLE (FinnGen);

E: T1D and RA (FinnGen); F: T1D and IBD (FinnGen)

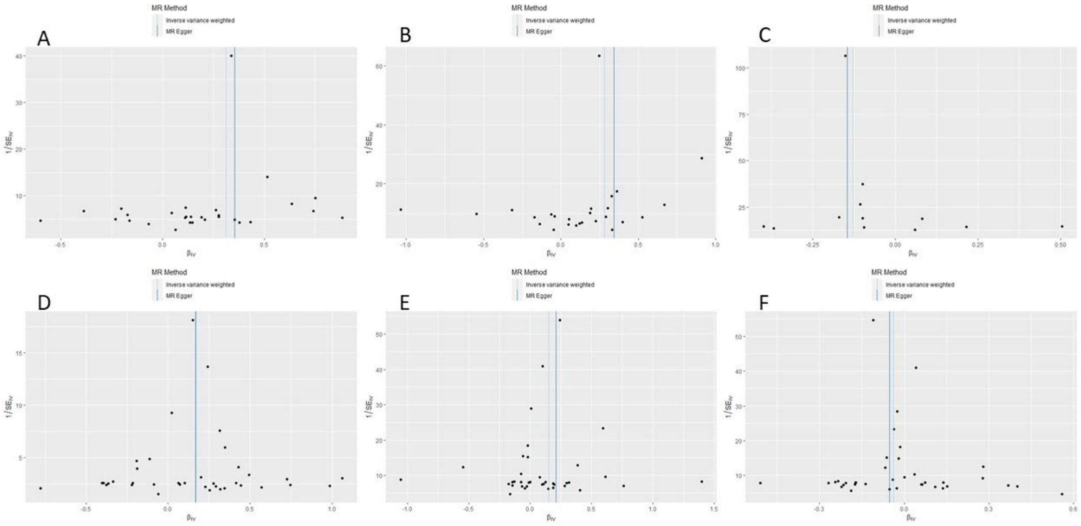

Supplementary Figure 3: Volcano plot of MR results

A: T1D and SLE (non-FinnGen); B: T1D and RA (non-FinnGen);

C: T1D and IBD (non-FinnGen); D: T1D and SLE (FinnGen);

E: T1D and RA (FinnGen); F: T1D and IBD (FinnGen)
